# Supplementary material for: Why multilingual, and how to keep it—An evolutionary dynamics perspective
Source: PLoS One. 2020 Nov 10;15(11):e0241980. doi: 10.1371/journal.pone.0241980 (PMC7654808; doi:10.1371/journal.pone.0241980)
Supplement: Attachment — Submitted filename: PLOS Evolution of Multilingualism Response Letter.pdf [file pone.0241980.s001.pdf]

Response letter for manuscript PONE-D-20-05223

Why multilingual, and how to keep it – An evolutionary dynamics perspective

submitted to PLOS ONE for publication

Dear Editor,

I would like to thank the reviewers for carefully reading the paper and providing valuable comments and suggestions. I have checked the manuscript and made thorough revisions based on their reviews. The following are my responses to the reviewers' comments and the corresponding changes I have made in the paper. My colleague, Professor Mark Hunacek, has helped me to copyedit the manuscript for language usage, spelling, and grammar. He is a well respected scholar, and has reviewed and written reports for over hundreds of math papers. Attached please find the revised paper with marked changes. A copy of the paper without marks is also submitted.

Thank you all very much!

Sincerely,

Zhijun Wu

Reviewer #1's General Comments:

In this MS, the author proposes an evolutionary dynamic model to study the evolution of multilingualism. The model consists two different parts. Concretely, the first one relates to selection of languages based on competition and the second one relates to circumstances when selection of languages is altered. Through theoretical analysis and numerical simulation, the results show that the stable co-existence of languages is possible and extinction can be prevented by choosing appropriate interventional strategies. Furthermore, the author gives the stability conditions of equilibrium states for different interventional strategies. Overall, this is a nice and well written paper, tackling an interesting question in the evolution of cooperation. The mathematical analysis is done carefully (although I have to admit that I am unable to check all formulas) and the results are presented in an understandable way.

Response to Reviewer #1's General Comments:

Thanks for carefully reading the paper and providing valuable comments and suggestions!

Reviewer #1's Comment #1:

The author respectively studies the evolutionary dynamics in competition-only and intervention-only populations. Furthermore, the author also combines these two and explores the stability of the equilibrium points. He/she make a combination of the systems in (3) and (6) to obtain a new system. Can the author explain in detail why the system can be described by equation 9.

Response to Reviewer #1's Comment #1:

Thanks for pointing this out. The original description was indeed unclear and should have provided a rationale behind the combination. In general, the combined form should be a convex combination of competition and intervention, i.e., the left-hand side of equation 9 should be a convex combination of the right-hand sides of equation 3 and 6. For simplicity, the paper

has focused on a simple form with an equal weight assigned to competition and intervention. To explain this, the first paragraph of the **Evolution with Competition and Intervention** section is now replaced with the following paragraph (highlighted in red):

Now the two types of games described above can be combined to obtain a complete model for the evolution of a bilingual population. A simple combination can be obtained by merging the systems in (3) and (6) into the following system:

$$\begin{cases} \dot{y}_A = y_A y_B[\lambda(P_A(y_A) - P_B(y_B)) + (1-\lambda)(\bar{P}_A(y_A) - \bar{P}_B(y_B))] \\ \dot{y}_B = y_B y_A[\lambda(P_B(y_B) - P_A(y_A)) + (1-\lambda)(\bar{P}_B(y_B) - \bar{P}_A(y_A))], \end{cases} \tag{9}$$

where $\lambda \in [0,1]$ is a scalar. If $\lambda = 1$, the system is reduced to the one in (3) with competition only. If $\lambda = 0$, it is reduced to the one in (6) with intervention only. If $\lambda \in (0,1)$, the system is in some sense a convex combination of competition and intervention, with more weight on competition when $\lambda > 0.5$ while more on intervention when $\lambda < 0.5$. For convenience, however, a simpler system, with an equal weight given to competition and intervention, will be considered in the following discussion:

$$\begin{cases} \dot{y}_A = y_A y_B(P_A(y_A) - P_B(y_B) + \bar{P}_A(y_A) - \bar{P}_B(y_B)) \\ \dot{y}_B = y_B y_A(P_B(y_B) - P_A(y_A) + \bar{P}_B(y_B) - \bar{P}_A(y_A)). \end{cases} \tag{10}$$

The original paragraph is the following (highlighted in blue):

We can combine the two types of games described above to obtain a complete model for the evolution of a bilingual population. We first make a combination of the systems in (3) and (6) to obtain the following system:

$$\begin{cases} \dot{y}_A = y_A y_B(P_A(y_A) - P_B(y_B) + \bar{P}_A(y_A) - \bar{P}_B(y_B)) \\ \dot{y}_B = y_B y_A(P_B(y_B) - P_A(y_A) + \bar{P}_B(y_B) - \bar{P}_A(y_A)). \end{cases} \tag{9}$$

Reviewer #1's Comment #2:

In the first part of the manuscript, the author studies the evolutionary dynamics of multilingualism in well-mixed populations, while in the latter part, he/she studies the evolutionary dynamics of multilingual on the 2D torus-shaped lattice. Here, I want to know the relationship between these two, because individuals in the lattice are not well-mixed.

Response to Reviewer #1's Comment #2:

In the first part of the manuscript, the evolutionary games and replication equations are all continuous models with populations assumed to be large and well-mixed. By well-mixed, it means that every individual can interact with all others in the population. The games played in the 2D simulation are discrete approximations to the continuous ones discussed in the first part of the manuscript, especially when the individual in each cell is allowed to interact with the individuals in all other cells in the lattice. In the latter case, the equilibrium states predicted by the 2D simulation should agree with those by the continuous models. However, when the interactions are restricted in a small neighborhood of each cell, the 2D simulation is indeed not done on a well-mixed population, and is not equivalent to a continuous model. On the other hand, the 2D simulation can show how a population behaves when the interactions are restricted, which a continuous model would not be able to reveal. To emphasize the point of carrying out the 2D simulation, a new paragraph is now inserted right after the third paragraph of the **Dynamic Simulation** section as highlighted in red in the following:

Recall that the population is assumed to be large and well mixed in the game models discussed in previous sections. This means that every individual should be able to interact with all others in the population. With such an assumption, the dynamic behaviors and especially the equilibrium

states of the population can be computed by directly solving a corresponding system of replicator equations. The 2D simulation described here can be considered as a discrete solution of the system of replicator equations especially when the individual in each cell of the lattice is allowed to interact with the individuals in all other cells. The equilibrium states of the population predicted by the 2D simulation should then agree with those by the continuous models. However, the 2D simulation is more flexible allowing restrictions on interactions. It can therefore be used to show how a population behaves when each individual interacts with only a selected group of other individuals, which a continuous model would not be able to reveal.

Reviewer #1's Comment #3:

The author study how multilingualism may evolve under the language competition and societal interventions. What does societal interventions usually mean here? What is the relationship between this kind of social intervention and some social incentive mechanisms? These social incentive mechanisms include punishment, reward, or exclusion such as Chen et al. PLoS Computational Biology, 14(7), e1006347 (2018), Liu et al. Nonlinear Dynamics, 97(1), 749-766, (2019).

Response to Reviewer #1's Comment #3:

Language competition includes influences from population sizes, social advantages, and economic preferences. For example, if language A has a larger population, more individuals would start using language A, etc. This interpretation agrees with previous assumptions such as by Abrams and Strogatz 2003. On the other hand, societal intervention is considered in this paper to be any influences from society or family to counter the arbitrary increase of one language but decrease in the other. Such influences may come from public policies, special education programs, family traditions, and immigration policies, etc. These distinctions between language competition and societal intervention are explained in the third paragraph of the **Introduction** section as shown in the following (original in blue; changed in red):

... The first part accounts for the selection of languages based on the competition for population and social or economic preferences. It assumes that the speakers are able to adjust their ways of using single or multiple languages, although in reality, it may take time and extra learning efforts. The second part relates to circumstances when the selection of languages is altered, for better or worse, by forces other than competition such as public policies, education, or family influences. Under these circumstances, the number of speakers of one language may be increased or decreased due to the impact of certain public policies or the migration of the speakers in or out of the population, etc.

To make the above discussions more clear, modifications are also made in the texts in sections **Evolution with Competition** and **Evolution with Intervention** (original in blue and changed in red):

In the first paragraph of the **Evolution with Competition** section:

Let $P_A(y_A)$ and $P_B(y_B)$ be the payoff functions for A and B speakers in a (y_A, y_B)-population, respectively, defined in terms of population percentages and social or economic preferences, with P_A increasing in y_A and P_B in y_B, meaning that the larger the population percentage of a language, the more benefit for the speaker of the language.

In the first paragraph of the **Evolution with Intervention** section:

Assume that the societal influences or interventions are implemented to counter the arbitrary increase or decrease of either language in a bilingual population. Let $\bar{P}_A(y_A)$ and $\bar{P}_B(y_B)$ be the payoff functions for A and B speakers in a (y_A, y_B)-population, respectively, defined in terms of societal influences and interventions, with $\bar{P}_A$ decreasing in y_A and $\bar{P}_B$ in y_B, meaning that the smaller the population percentage of a language, the more incentive or less penalty the speaker

of the language receives. Here, incentive means more benefit from the society such as more work opportunities or public supports, etc., and penalty means less advantage in the society such as less access to public education or discouragement from the public, etc.

As for reward or punishment, they may not be directly implemented for language uses except in extreme cases. However, they are always implicitly implied by the social or economic impacts. The reward and punishment methods proposed by Chen et al. 2018 and Liu et al. 2019 may be employed for future research on interventional strategies. The two references are therefore added to the paper and cited in the end of the third paragraph of the **Discussion** section as highlighted in red in the following:

While the parameters used in the proposed models are key to determining general dynamic behaviors of multilingual populations, more parameters may be introduced, and additional mechanisms such as the reward and punishment schemes discussed in [23,24] may be employed.

Reviewer #1's Comment #4:

The figures in the manuscript should be more exquisite. Concretely, some circles are incomplete. In addition, stable and unstable equilibrium points cannot be distinguished. What does the axis in Figure 5 represent?

Response to Reviewer #1's Comment #4:

The figures are re-generated now, with equilibrium points all marked with a half circle. The stable equilibrium is indicated by two arrow lines pointing to it, while the unstable one by two arrow lines leaving it. Each of the graphs in Fig 5 is a 75×75 2D lattice. The x-axis and y-axis of the graph represent the 75 units of the lattice in the horizontal and vertical directions, respectively. (This sentence is now added to the caption of Fig 5.)

Reviewer #1's Comment #5:

There are some grammatical problems in the manuscript that need to be further checked. Besides, the format of some references needs to be adjusted, for example, [8], [31-32], [34], [36].

Response to Reviewer #1's Comment #5:

The formats of all the references are now adjusted according to the guidelines of the journal. An American colleague Professor Mark Hunacek has helped to read through the paper and corrected all grammatical problems in the manuscript.

Reviewer #2's General Comments:

The paper investigates the evolution of bilingualism in the framework of evolutionary game theory. The author constructs an evolutionary game-theoretical model between two languages by considering not only the inner competition between them, as Abrams and Strogatz do (Nature 424: 900, 2003), but also the social interventions that are imposed by the external factors such as public policies, education, or family influences. By combining competition with intervention, they show both languages can be stably coexisted in the well-mixed populations. Besides, the author also performs computer simulations to explore the evolutionary dynamics on spatial lattices.

In general, I think this is a good paper. Particularly, it complements the results previously found by Abrams and Strogatz, that is, the evolutionary competition between two languages leads to the extinction of either language. The findings of this paper clearly show us that how bilingualism may evolve under the influences of both competition and intervention.

Response to Reviewer #2's General Comments:

Thanks for carefully reading the paper and providing valuable comments and suggestions!

Reviewer #2's Comment #1:

The title of the manuscript is "Why Multilingual, and How to Keep It – An Evolutionary Dynamics Perspective". But I find that the author merely investigated the dynamic behaviors of bilingual population. If the author insists on usage of the present title, I think the author should extend the bilingual model to multilingual model, and mainly study the dynamic behaviors of multilingual population.

Response to Reviewer #2's Comment #1:

The purpose of this paper is to propose a general theoretical framework for the study of evolution of multilingual populations as stated in the title and set up in the **Introduction** section. To keep the description simple and easy to follow, the proposed models are defined and analyzed only with bilingual populations, but they are not limited to bilingual populations. They can actually be extended straightforwardly to general multilingual populations, as given in Appendix E. Therefore, I would still like to keep the original title, and add some comments in the paper for smooth transitioning of the discussions from multilingual to bilingual populations. First, in both **Abstract** and **Introduction**, a statement is now modified as shown in the following (highlighted in red) to make clear that multilingual populations are considered in general in the paper, while bilingual populations are focused in particular.

In this paper, a mixed population of multilingual speakers and bilingual speakers in particular is considered, with multilingual defined broadly as zero, limited, or full uses of multiple languages or dialects, and an evolutionary dynamic model for its development and evolution is proposed.

Second, at the beginning of the **Evolutionary Models** section, the following sentence (highlighted in red) is added for the start of the focus on bilingual populations.

To keep the description simple and easy to follow, consider a bilingual population, the simplest yet most common form of multilingualism.

At the beginning of the **Dynamic Analysis** section, the following sentence (highlighted in red) is also added for the continuation of the discussion on bilingual populations.

Continue the assumption of bilingual populations and consider their possible dynamic behaviors based on the models described in previous sections.

Third, in both **Introduction** and **Discussion**, a paragraph as shown in the following (highlighted in red) is attached to emphasize that the models proposed in the paper can be extended straightforwardly to general multilingual populations as shown in Appendix E, although the discussions have been focused on bilingual populations for easy to explain and analyze.

To keep the description simple and easy to follow, the proposed models are introduced with and analyzed on bilingual populations only, but they are not limited to bilingual populations. They can actually be extended straightforwardly to general multilingual populations, as given in Appendix E. In order to make the paper more accessible, efforts have been made to keep the discussions from being too technical, with more formal definitions, theorems, and especially proofs all provided as appendices in the end of the paper. Nonetheless, these appendices are considered to be necessary contents as well for supporting the results and conclusions presented in the paper.

Reviewer #2's Comment #2:

It seems to me that the author assume that language competition and social intervention have equal impact in the evolution of bilingualism due to the same weights assigned to them in Eq. (9). This maybe a too strong assumption in my opinion. I think it is necessary to explore what

happens if they have different impacts in the evolution of bilingualism? Is the present conclusion robust against such changes? If the answer is "No", what are the new results?

Response to Reviewer #2's Comment #2:

Thanks for pointing this out. The original description was indeed unclear and should have provided a rationale behind the combination. In general, the combined form should be a convex combination of competition and intervention, i.e., the left-hand side of equation 9 should be a convex combination of the right-hand sides of equation 3 and 6. For simplicity, the paper has focused on a simple form with an equal weight assigned to competition and intervention. To explain this, the first paragraph of the **Evolution with Competition and Intervention** section is now replaced with the following paragraph (highlighted in red):

Now the two types of games described above can be combined to obtain a complete model for the evolution of a bilingual population. A simple combination can be obtained by merging the systems in (3) and (6) into the following system:

$$\begin{cases} \dot{y}_A = y_A y_B[\lambda(P_A(y_A) - P_B(y_B)) + (1-\lambda)(\bar{P}_A(y_A) - \bar{P}_B(y_B))] \\ \dot{y}_B = y_B y_A[\lambda(P_B(y_B) - P_A(y_A)) + (1-\lambda)(\bar{P}_B(y_B) - \bar{P}_A(y_A))], \end{cases} \tag{9}$$

where $\lambda \in [0,1]$ is a scalar. If $\lambda = 1$, the system is reduced to the one in (3) with competition only. If $\lambda = 0$, it is reduced to the one in (6) with intervention only. If $\lambda \in (0,1)$, the system is in some sense a convex combination of competition and intervention, with more weight on competition when $\lambda > 0.5$ while more on intervention when $\lambda < 0.5$. For convenience, however, a simpler system, with an equal weight given to competition and intervention, will be considered in the following discussion:

$$\begin{cases} \dot{y}_A = y_A y_B(P_A(y_A) - P_B(y_B) + \bar{P}_A(y_A) - \bar{P}_B(y_B)) \\ \dot{y}_B = y_B y_A(P_B(y_B) - P_A(y_A) + \bar{P}_B(y_B) - \bar{P}_A(y_A)). \end{cases} \tag{10}$$

The original paragraph is the following (highlighted in blue):

We can combine the two types of games described above to obtain a complete model for the evolution of a bilingual population. We first make a combination of the systems in (3) and (6) to obtain the following system:

$$\begin{cases} \dot{y}_A = y_A y_B(P_A(y_A) - P_B(y_B) + \bar{P}_A(y_A) - \bar{P}_B(y_B)) \\ \dot{y}_B = y_B y_A(P_B(y_B) - P_A(y_A) + \bar{P}_B(y_B) - \bar{P}_A(y_A)). \end{cases} \tag{9}$$

Reviewer #2's Comment #2:

With reference to the computer simulations on evolutionary game dynamics in spatial networks, I suggest citing the following papers: Phys. Rev. E 78, 051120 (2008) and Sci. Rep. 2, 740 (2012), wherein the authors used computer simulations for exploring the spatial dynamics of evolutionary games.

Response to Reviewer #2's Comment #2:

The suggested two papers are added to the references and cited in the text. Thanks!

Reviewer #3's General Comments:

My major concern with the paper is as follows: I think it is too theoretical to be published in a general journal such as PLOS ONE. In the current form, I would suggest to submit it to another journal where this kind of article type is common. Moreover, I think that there is a huge gap between empirical data and the model's predictions. I understand that a model with such a high degree of abstraction from reality is hard to be checked against empirical data. However,

here even a preliminary attempt is missing (as the author mentioned himself in the Discussion section). Finally, I think there are also some at least questionable aspects of the model definition with respect to how languages are actually acquired, learned or competitively/cooperatively used in real societies. It is not clear to me that the model particularly defines language use. What are the language-specific aspects? It seems to me as if A and B could also be other cultural or social traits and customs, which can be adopted or abandoned, used to initiate competition or cooperation, and supported by societal interventions.

Response to Reviewer #3's General Comments:

Thanks for carefully reading the paper and providing valuable comments and suggestions! The modeling of multilingual populations has become an active interdisciplinary research area especially since the publication of the Abrams and Strogatz paper in 2003. It involves research in (1) language development, (2) multilingual education, (3) complex social systems, (4) mathematical modeling, (5) statistical surveys, (6) dynamic simulation, and (7) stability analysis, etc. The topics of the submitted paper are related to (4), (6) and (7), which are indeed more theoretical but not out of the scope. The papers on multilingual modeling have been published on journals like Nature, Science, Physics A, PNAS and contributed by researchers from diverse disciplines including applied mathematics, theoretical physics, and linguistic and social sciences. PLOS ONE is a multidisciplinary journal with readers in a broad range of disciplines. I would therefore love to see papers on multilingual modeling published on PLOS ONE, either theoretical or experimental.

A great concern in the field of multilingual modeling is the paradox between language death and language co-existence. On one hand, Abrams and Strogatz 2003 showed with their model and data that when two languages compete, one will eventually die while the other will sustain. While this is partially true for some language populations, on the other hand, we do see some populations with multiple languages co-existing, and languages often co-exist in various multilingual forms. The submitted paper is intended to address this concern by proposing a more complete model than the one by Abrams and Strogatz with which the conditions can hopefully be found for both language death and co-existence. An evolutionary dynamic model is proposed in the paper to account for influences from both language competition and societal intervention on multilingual development. Careful analysis is done to show in what circumstances and under what conditions languages co-exist or die otherwise. Computer simulation is also conducted to demonstrate possible geographical or demographical impacts on multilingual development. The work is theoretical, but addresses a practical concern and a core issue in the current field of multilingual modeling.

The paper is indeed a little bit more mathematical, even after the formal definitions, theorems, and proofs are moved to the appendices. After all, the issue addressed by the paper is a mathematical one, and does require rigorous justification and analysis. On the other hand, the evolutionary game theory which the whole work is based on is not really restricted only to the field of mathematics. It has been applied to a broad range of scientific fields including biology, economics, and political sciences. For example, it has been adopted as a major analysis tool for animal behaviors in evolution biology and ecology for years. Therefore, I would consider this paper as a modest mathematical contribution I could probably make for multilingual modeling and at the same time, as a piece of work to demonstrate the potential application of evolutionary game theory to language evolution, which hopefully makes the mathematical abstraction of the paper more worthwhile.

As described in the paper, when the payoffs $P_A, P_B, \bar{P}_A, \bar{P}_B$ are defined by the functions in (16) and (17), the proposed model is really a direct extension from the Abrams-Strogatz model, which has been verified on a large set of language data, with α determined to be approximately equal to 1.31 ± 0.25. In the proposed model, the part for language competition is really an Abrams-Strogatz equation with $1 < \alpha \leq 2$ and the part for societal intervention is an Abrams-Strogatz

equation with $0 \leq \alpha < 1$. Therefore, the proposed model does have some realistic basis through its connection to Abrams-Strogatz, and is not completely beyond reality. Hopefully, it will be better determined and refined with more language data in future work.

Indeed this paper has not discussed in detail how the language is acquired, learned, and used, which may in fact relate directly to what language strategies really mean and how they can be changed. There are some obvious questions related to this issue. For example, what does it mean that an individual uses language A with 40% frequency? Is only reading language A without speaking counted as using language A? What happens if an individual can never learn a second language? How long does it take for an individual to change his/her language use from one frequency to another? These are legitimate concerns, but may require more linguistic characterizations on language learning and communication, and will hopefully be addressed in future work. To make this issue clear, in the revised version of the paper, the following paragraph (highlighted in red) is added in the **Discussion** section:

Note that this paper has not discussed in detail how the language is acquired, learned, and used, which may in fact relate directly to what language strategies really mean and how they can be changed. There are some obvious questions related to this issue. For example, what does it mean that an individual uses language A with 40% of frequency? Is only reading language A without speaking counted as using language A? What happens if an individual can never learn a second language? How long does it take for an individual to change his/her language use from one frequency to another? These are legitimate concerns, but may require more linguistic characterizations on language learning and communication, and will hopefully be addressed in future work.

Finally, the models proposed in the paper may indeed be applied to other social systems, but the corresponding social traits have to share similar features with language. For example, an important feature of language is that the use of a language increases with increasing size of the population of the language unless there are some societal inventions. Similar to this, the social behavior for fashion following also prefers more popular fashions. However, the social activities of choosing among religious beliefs may not always follow the beliefs of the majorities.

Reviewer #3's Comment #1:

In line 24/25: "It assumes that the speakers are free to choose among available languages." I think this is a unrealistic assumption. Speakers mostly learn a new language due to their (new) social environment, for example as a result of migration. They are mostly forced to learn the new local language to be able to participate in the social environment. Here it sounds as if speakers can change their language from one day to the other as a free choice.

Response to Reviewer #3's Comment #1:

The assumption is indeed too strong. To be more accurate, the quoted sentence is now changed to "It assumes that the speakers are able to adjust their ways of using single or multiple languages, although in reality, it may take time and extra learning efforts.". The assumption is strong especially if the languages are very different. However, it is not completely unrealistic if the languages are similar, especially if they are similar dialects of the same language. For example, in the city of Xian, Shaanxi Province and Jinan of Shandong Province of China, the local residents can freely speak both Mandarin and their local Chinese dialects, yet the social status and popularity of Madarine are still more or less very different from those local dialects, and the increasing use of Mandarin is a result of the public policy.

Reviewer #3's Comment #2:

In line 81: "... that the larger the population percentage of a language, the more competitive the speaker of the language." Why are they more competitive? It there empirical evidence for

that? Shouldn't the payoff be dependent either i) on (mutual) intelligibility ii) or (probably more important here) on the social and economic benefits of using a particular language? I am not convinced that majority alone is sufficient for a language to be more competitive per se.

Response to Reviewer #3's Comment #2:

Sorry, the sentence should be "... that the larger the population percentage of a language, the more competitive the language", meaning the language provides more benefits because the speakers can communicate with a larger percentage of population. To make it more clear, the sentence is now changed to "... that the larger the population percentage of a language, the more benefit the language provides." This property is well represented by the monotonic increasing property of the payoff functions $P_A(y_A)$ and $P_B(y_B)$, which has been justified on a large set of language data in Abrams and Strogatz 2003, where P_A and P_B are defined as functions of $y_A^{\alpha-1}$ and $y_B^{\alpha-1}$, with $\alpha = 1.31 \pm 0.25$. The payoffs indeed also depend on social and economic benefits as they are represented by parameters s_A and s_B in P_A and P_B.

Reviewer #3's Comment #3:

In line 107/108: "... meaning that the smaller the population percentage of a language, the more incentive the speaker of the language receives." What does the payoff actually stands for? Here it represents the incentive a speaker receives. Is this also the case for the competitive scenario? It is not clear at all what the payoff stands for in general. Maybe the author makes this point more clear to the reader.

Response to Reviewer #3's Comment #3:

To make it more specific, at the beginning of the paragraph of the quoted sentence, the following sentence is added "Assume that the societal influences or interventions are implemented to counter the arbitrary increase or decrease of either language in a bilingual population." Then, the quoted sentence is expanded as follows: "... meaning that the smaller the population percentage of a language, the more incentive or less penalty the speaker of the language receives. Here, incentive means more benefit from the society such as more work opportunities or public supports, etc., and penalty means less advantage in the society such as less access to public education or discouragement from the public, etc."

Reviewer #3's Comment #4:

Again, considering the high degree of abstraction of the model from the real world, it would be good to see how the model predicts real world phenomena. In the introduction, the author points to the many places in the world where multilingualism exists. But is there empirical evidence that interventions are the (unique) reason for its existence/perpetuation? Is it possible to get data about language competition and societal interventions, etc, that the model can be tested against?

Response to Reviewer #3's Comment #4:

At this point, only general observations on societal interventions are available as described in the **Introduction** section. The revival of the Hebrew in the 19th century is an obvious case, which would be impossible if without the community's wills, the family influences, and the impacts of the historical social movements. The popularization of Mandarin in China is a direct result of the government policy of using Mandarin as the standard official Chinese. It is believed that if the official language were changed to another dialect other than Mandarin, the popularity of Mandarin would have been dramatically reduced, especially in southern China. Of course, it would indeed be interesting to collect a large set of language data from specific multilingual populations and see how the parameters in the proposed models can be determined for both language competition and societal intervention. Unfortunately, there is no such data

ever collected yet.

Reviewer #3's Comment #5:

Spelling: Abstract: and playing −> and is playing; Abstract: accounts for selection −> accounts for the selection; In general: the article 'the' is dropped very often: (the) selection, (the) evolution, etc; in line 56: in home −> at home

Response to Reviewer #3's Comment #5:

All revised. An American colleague Professor Mark Hunacek has helped to double check the English in the paper.

The paper has been revised to meet the manuscript requirements by PLOS ONE. The formats for the references have been adjusted by following the guidelines of the journal. The original uses of the first-person pronoun "we" have been changed throughout the paper.
